# Tooth loss impairs cognitive function in SAMP8 mice by aggravating pyroptosis of microglia via the cGAS/STING pathway

**DOI:** 10.3389/fnagi.2025.1628520

**Published:** 2025-08-22

**Authors:** Xu Sun, Yunping Lu, Jiangqi Hu, Shixiang Meng, Xiaoyu Wang, Qingsong Jiang

**Affiliations:** Department of Prosthodontics, Beijing Stomatological Hospital, School of Stomatology, Capital Medical University, Beijing, China

**Keywords:** Alzheimer’s disease, cognitive dysfunction, tooth loss, cGAS/STING, GSDMD, SAMP8

## Abstract

**Introduction:**

Alzheimer’s Disease (AD) is a common neurodegenerative disease among the elderly population. It has been posited that the onset and progression of AD are influenced by a combination of various factors. Occlusal support loss due to tooth loss has been reported to be a risk factor triggering cognitive dysfunction. This study aimed to investigate the relationship between tooth loss and cognitive dysfunction and illustrate the role of pyroptosis in advancing Alzheimer’s disease.

**Methods:**

Male 5-month-old senescence-accelerated mouse strain P8 (SAMP8) mice were divided into two groups (n = 6): the S (sham-operated) and TL (tooth loss) groups. We assessed spatial memory ability using the Y-maze and Novel Object Recognition (NOR) tests. In addition, we performed pathological and molecular biological assessments of the hippocampus to evaluate pyroptosis-related indicators and changes in cGAS/STING. We further verified the correlation between the two *in vitro*.

**Results:**

The pathological section staining revealed an upregulation of GSDMD, a target protein of pyroptosis, and abnormal activation of the cGAS/STING pathway, particularly in microglia, after tooth loss. *In vitro*, we demonstrated that the BV2 microglia knockdown STING group improved the inflammatory cascade response and down-regulated the pyroptotic features.

**Discussion:**

These data suggest that the occlusal support loss due to tooth loss induces pyroptosis-related protein deposition, which may be intimately associated with the cGAS/STING signaling pathway. This provides new insights into the treatment and prevention of oral health and cognitive behavioural disorders in the elderly population.

## 1 Introduction

Alzheimer’s disease (AD) is a progressive neurodegenerative disorder that occurs in the elderly population. This disorder has an insidious onset and is characterized by cognitive impairment and non-cognitive neuropsychiatric symptoms (Graff-Radford et al., 2021). Tooth loss, a common oral disease, affects the quality of life of the elderly population as much as AD (Xu et al., 2023; Aminoshariae et al., 2024). Numerous studies have shown that tooth loss was significantly associated with cognitive impairment, dementia, and AD (Li et al., 2023; Wang et al., 2022; Nakamura et al., 2021). As early as 2002, Watanabe et al. found that tooth loss resulted in decreased hippocampal activity and cognitive function in aged SAMP8 mice (Watanabe et al., 2002). Cerutti-Kopplin et al. conducted a meta-analysis of literature reviews and found that subjects with fewer than 20 functional teeth had a 26% increased risk of cognitive decline and a 22% increased risk of dementia (Cerutti-Kopplin et al., 2016). The consequences of tooth loss extend beyond the domain of cognitive function. Found that motor skills and decision-making behaviors were impaired in rats (Xu et al., 2015). Dibello et al. proposed the concept of oral frailty, which is a decline in oral function with a coexisting decline in cognitive and physical functions (Dibello et al., 2021).

Our group has been engaged in exhaustive research in this area for a considerable number of years. As has been documented, the loss of teeth has been shown to result in diminished cerebral blood perfusion and significant microglia activation, which, in turn, can lead to cognitive impairment (Luo et al., 2019; Pang et al., 2020). And related changes, such as neuroinflammation in the brain of SAMP8 mice after tooth loss, were found (Sun et al., 2024).

Microglia have been identified as pivotal mediators of neuroinflammation and the immune response following central nervous system (CNS) injury. Furthermore, they have been recognised as the primary cell type in which pyroptosis occurs in the CNS (Ding et al., 2022). Pyroptosis, a discovered form of programmed cell death, has been characterized by gasdermin D (GSDMD)-mediated rupture of cellular integrity, osmotic swelling, and release of pro-inflammatory factors (Cao et al., 2023). The N-terminal domain of GSDMD (GSDMD-N) causes plasma membrane perforation, releasing abundant proinflammatory cytokines, spreading inflammatory signals, and exacerbating the onset and progression of neurodegenerative diseases (Zhang et al., 2019; Li et al., 2021). The present study explores the potential of exploring the pathogenesis of AD from the perspective of the pyroptosis pathway as a novel research direction.

Cyclic GMP-AMP synthase (cGAS) is a crucial cytoplasmic DNA receptor that recognizes endogenous and exogenous double-stranded DNA (dsDNA) (Samson and Ablasser, 2022; Decout et al., 2021). Upon binding to dsDNA, cGAS forms a 2:2 dimer, inducing a change in the active site and catalyzing the synthesis of cyclic adenosine guanosine monophosphate (cyclic AMP) and guanosine triphosphate (GTP), as well as 2′,3′-cyclic GMP-AMP (2′,3’-cGAMP) (Sun and Hornung, 2022). cGAMP functions as a second messenger that binds to the endoplasmic reticulum (ER). cGAMP recruits and activates TANK-binding kinase 1 (TBK1) at the carboxyl terminus of activated STING, which is subsequently involved in expressing a range of inflammation-related genes (Dvorkin et al., 2024). It is noteworthy that the available evidence indicates that the activation of cGAS-STING leads to the induction of reactive microglial transcriptional states, neurodegeneration, and cognitive decline (Gulen et al., 2023).

Although the mechanisms underlying the role of tooth loss in cognitive dysfunction have been the focus of study for decades, the interaction between the cGAS-STING axis and microglia pyroptosis after tooth loss remains to be determined. The present study, therefore, sought to evaluate the effect of tooth loss on spatial performance and cognitive function in SAMP8 mice after tooth loss and to investigate whether the cGAS/STING pathway plays a key role in cognitive dysfunction induced by microglia pyroptosis.

## 2 Materials and methods

### 2.1 Animals

Five-month-old male senescence-accelerated mouse strain P8 (SAMP8) mice weighing 45-55 g were purchased from Beijing Huafukang Biotechnology Co. (Chica) and transported to the animal housing facility at Beijing Stomatological Hospital. Mice were kept in standard cages under standard conditions (room temperature of 25°C, 40% humidity) with a 12 h light/dark cycle for 2 months. Mice had free access to food pellets and water. All animal study protocols followed the National Institutes of Health guidelines for the care and use of laboratory animals and were approved by the ethics committee of Capital Medical University (IACUC: AEEI-2022-026).

### 2.2 Group assignment and surgery method

After 1 week of adaptation to the new environment, SAMP8 mice (*n* = 12) were randomly divided into two groups of six animals (S group [sham operation group] and TL group [bilateral maxillary tooth loss]). The mice were anesthetized via intraperitoneal injection of 10% chloral hydrate (400 mg/kg). All bilateral molars in the maxillary region were removed in the TL group. The S group was subjected to artificially caused gingival damage to the bilateral maxillary molars but no tooth extraction. Finally, each SAMP8 mouse was injected with buprenorphine (50 mg/g) intramuscularly to alleviate suffering during surgery. Behavioral tests, including the Y-maze and novel object recognition, were conducted 8 weeks after the animal model was established. Following the completion of behavioral experiments, the mice were immediately euthanized, and hippocampal tissue was collected.

### 2.3 The Y-maze test

The Y-maze consists of three arms of 30, 8, and 15 cm in length, width, and height, respectively, with an angle of 120° between the two arms. Mice were placed at the end of one of the three arms and the order in which they entered each arm within 5 min was recorded. Entry of the mice into each arm is standardized as complete entry of all limbs. Mice sequentially entering a different arm than the previous two were considered a correct alternation, and the percentage of spontaneous alternations was used to assess the memory capacity of the mice. Spontaneous alternations = (number of correct alternations) / (total number of arm entries − 2) × 100%. For spontaneous alternation experiments, behavioral testing with a cycle of 1 day can begin after 30 min of environmental adaptation.

### 2.4 Novel object recognition (NOR)

The NOR experiment was divided into training and test phases, and a 3-day environmental acclimatization period was required before the training phase. In the training phase, two equivalent A objects were placed on opposite sides of the test box, with the objects about 10 cm away from the wall of the box; the mice were then placed in the test box and removed after 5 min of free exploration. During the test phase, an A object (familiar object) was randomly replaced by a B object (novel object) in the test box, and the position remained unchanged; the mouse was then placed in the test box and removed after 5 min of free exploration. During the test phase, the mice’s exploration times for familiar and novel objects were recorded separately, and the recognition index (RI) was calculated to assess the memory ability. The RI was calculated as follows: RI = (new object exploration time − old object exploration time)/(new object exploration time + old object exploration time) × 100%. For the NOR experiment, an adaptation period (3 days), training period (1 day), and testing period (1 day) are required.

### 2.5 Hippocampus collection

Following behavioral experiments, SAMP8 mice were anesthetized by intravenous administration of pentobarbital sodium (15–40 mg/kg) and euthanized. Right and left brain tissues were subsequently extracted. Some were frozen at −80°C for subsequent molecular biology experiments, some were fixed in 2.5% glutaraldehyde stationary solution for transmission electron microscopy, and some were fixed in 4% paraformaldehyde for 24 h and then subjected to gradient alcohol dehydration. The brain tissue was then embedded into paraffin for subsequent histopathological testing.

### 2.6 Cell culture

The murine microglial BV-2 cell line (cat NO. CL-0493) was obtained from Wuhan Pricella Biotechnology Co., Ltd. (Wuhan, China). BV-2 cells were cultured in DMEM medium (11965092, Gibco, USA) supplemented with 10% fetal bovine serum (FBS, 1693361, Gibco, USA), 100 U/mL penicillin, and 100  U/mL streptomycin. All cells were incubated in a humidified atmosphere of 5% CO2 at 37°C.

### 2.7 Small interfering RNA (siRNA) transfection

siRNA, against mouse STING and negative control, were designed and synthesized by Sangon Biotech (Shanghai, China). siRNA transfection was conducted according to the manufacturer’s instructions. Briefly, the transfection reagent Lipofectamine RNAiMAX (13778100, Invitrogen) and siRNA were mixed in Opti-MEM. The cells were then changed to normal medium and continued culturing for 24–48 h before following the experiments.

### 2.8 Immunohistochemistry

According to a standard nonbiotinylated protocol, the paraffin-embedded tissue sections were deparaffinized and rehydrated. The sections were then incubated overnight at 4°C with the primary antibodies, including rabbit anti-TFAM antibody (1:1000, A3173 ABclonal, China). Then, they were incubated for 20 min at 37°C with anti-rabbit IgG before being treated with diaminobenzidine (DAB) for approximately 1 min at room temperature. After the sections were sealed with neutral balsam (Biosharp, China), the sections were ready for light microscopy (Olympus/BX61, Japan). For the histomorphometric analysis, we selected three regions of interest (ROIs) in the hippocampus and measured the area of the ROI, the area of positive cells in the ROI, and the IOD (the area of positive cells in the ROI/ the area of the ROI).

### 2.9 Immunofluorescence

Following the standard instructions, we performed immunofluorescence staining of paraffin-embedded tissue sections using the Treble-Fluorescence immunohistochemical mouse/rabbit kit (RS0035, Immunoway, USA). The sections were incubated overnight at 4°C with the primary antibody, including rabbit IBA-1 antibody (1:1000, ab178846, Abcam, USA), rabbit GFAP antibody (1:5000, ab7260, Abcam, USA), rabbit NeuN antibody (1:100, ab177487, Abcam, USA), rabbit STING/TMEM173 antibody (1:100, A3575 ABclonal, China) and mouse GSDMD antibody (1:200, 66387-1-lg, Proteintech, China).

BV2 cells were seeded on glass coverslips in 6-well plates. After various treatments, cells were fixed with 4% paraformaldehyde for 20 min at room temperature and then washed three times with PBS. After permeabilization with 0.1% Triton X-100/PBS for 15 min, cells were rinsed with PBS, blocked with PBS containing 3% BSA for 1 h at room temperature, and then incubated with mouse GSDMD (1:200, 66387-1-lg, Proteintech, China) primary antibodies at 4°C overnight. The subsequent steps are the same as for paraffin sections.

### 2.10 Nissl staining

The paraffin-embedded tissue sections were deparaffinized and rehydrated. Consequently, the sections were stained with 1% Cresyl Violet staining solution (G1430, Nissl Stain Kit, Solarbio, China) in a 56°C thermostat for 1 h. The sections were dehydrated by fractional xylene and ethanol, and finally sealed with neutral gum, dried, and observed under an optical microscope for photography. Brain tissue was observed under a microscope after drying and analyzed via imaging.

### 2.11 Cell viability assay

Viable and non-viable cells were detected using Calcein/PI Cell Viability/ Cytotoxicity Assay Kit (C2015M, Shanghai Beyotime Biotechnology Co., Ltd.). The cells in each of the experimental groups were observed under a fluorescence microscope (Olympus/BX61, Japan).

### 2.12 Transmission electron microscope (TEM)

We took 1 mm x 1 mm x 3 mm hippocampal CA1 mid-section tissue and fixed it with 2.5% glutaraldehyde fixative before making electron microscope sections. The sections were embedded in epoxy resin and double-stained with uranyl acetate and lead citrate on an ultrathin microtome. The microstructure of the hippocampal tissue was observed and photographed under a JEM1200EX transmission electron microscope.

### 2.13 Enzyme-linked immunosorbent assay (ELISA)

Mouse hippocampal tissues and cells were lysed in Pierce RIPA Buffer was added to prevent protein degradation. The protein concentration of the lysate was measured using the Pierce BCA Protein Assay (23227, Thermo Fisher Scientific, USA) to normalize the 2′3′-cGAMP concentrations measured by ELISA according to the manufacturer’s instructions (EIAGAMP, Thermo Fisher Scientific, USA).

### 2.14 Real-time polymerase chain reaction

SAMP8 mice were sacrificed, and brain tissue was isolated from the hippocampus on ice. Then, the hippocampi were frozen in liquid nitrogen and stored at −80°C. As for bv2 cells, after blowing the adherent cells, the culture medium was aspirated and centrifuged to collect the cells for subsequent RNA extraction. According to the manufacturer’s instructions, total RNA was extracted with an Ultrapure RNA Kit (CWBIO, China). First-strand cDNA synthesis was performed using an All-in-one 1st Strand cDNA Synthesis SuperMix Kit (Novoprotein, China). The results were analyzed following the 2^−ΔΔCt^ method using *β*-actin as the control. MagicSYBR Mixture (CWBIO, China) with a Bio-Rad CFX Connect real-time system (CFX Connect Optics Module, Bio-Rad) was used. All RT–PCR experiments were repeated three times.

The primer sequences used were as follows:

Mouse GSDMD: 5′-CCATCGGCCTTTGAGAAAGTG-3′ and 5′-ACACATGAATAACGGGGTTTCC-3′; mouse cGAS: 5′-AGGAAGCCCTGCTGTAACACTTCT-3′ and 5′-AGCCAGCCTTGAATAGGTAGTCCT-3′; mouse STING: 5′-GGCGTCTGTATCCTGGAGTA-3′ and 5′-TAGACAATGAGGCGGCAGTTAT-3′. Mouse beta-actin (*β*-Actin): 5′-AGATTACTGCTCTGGCTCCTAGC-3′ and 5′-ACTCATCGTACTCCTGCTTGCT-3′.

### 2.15 Western blot analysis

As with RT-PCR, we performed Western blot analysis using SAMP8 mouse hippocampal tissue and BV2 cells, respectively. Proteins were extracted with an Applygen total protein extraction kit. The protein concentration was normalized using Coomassie brilliant blue G-250 staining. Equal amounts of proteins were separated by SDS–PAGE on a 10% polyacrylamide gel, and the proteins were transferred to PVDF membranes. After blocking with 0.1% TBST containing 5% nonfat milk at room temperature for 2 h. Primary antibodies against GSDMD (1:5000, 66387-1-lg, Proteintech, China), GSDMD-N (1:1000, YT7991 Immunoway, USA), cGAS (1:1000, A8335 ABclonal, China), STING/TMEM173 (1:2000, A3575 ABclonal, China), TBK1/NAK (1:1000, 3504 Cell Signaling Technology, USA), p-TBK1/NAK (1:1000, 5483 Cell Signaling Technology, USA), NLRP3 (1:1000, 15101 Cell Signaling Technology, USA), IL-18 (1:1000, 57058 Cell Signaling Technology, USA) and GAPDH (1:1000, 2118 Cell Signaling Technology, USA) were added for overnight incubation. The membrane was rinsed with 0.1% TBST three times for 10 min each and incubated with a secondary antibody (1:3000, 7074S, Cell Signaling Technology, USA) at room temperature for 1 h. Color development was performed using an ECL kit. Images were acquired using the Bio-Rad Imager and analyzed with ImageJ to measure the integrated optical density (IOD) values of specific bands.

### 2.16 Statistical analysis

Statistical analysis was performed using SPSS Statistics V17.0 software (SPSS Inc.). Normality was tested using the Shapiro–Wilk test. Statistical significance was determined using statistical tests detailed in both the figure legends and the Results section. These tests included the following: unpaired two-tailed t-test (when normally distributed by the Shapiro–Wilk test and the SDs are not different), unpaired two-tailed t tests with Welch’s correction (when normally distributed but SDs were unequal), and Mann–Whitney U test (when not normally distributed). The statistical significance level was 0.05. All graphs were made using GraphPad Prism 8.

## 3 Results

### 3.1 Effect of tooth loss on body weight in SAMP8 mice

**Figure 1:**
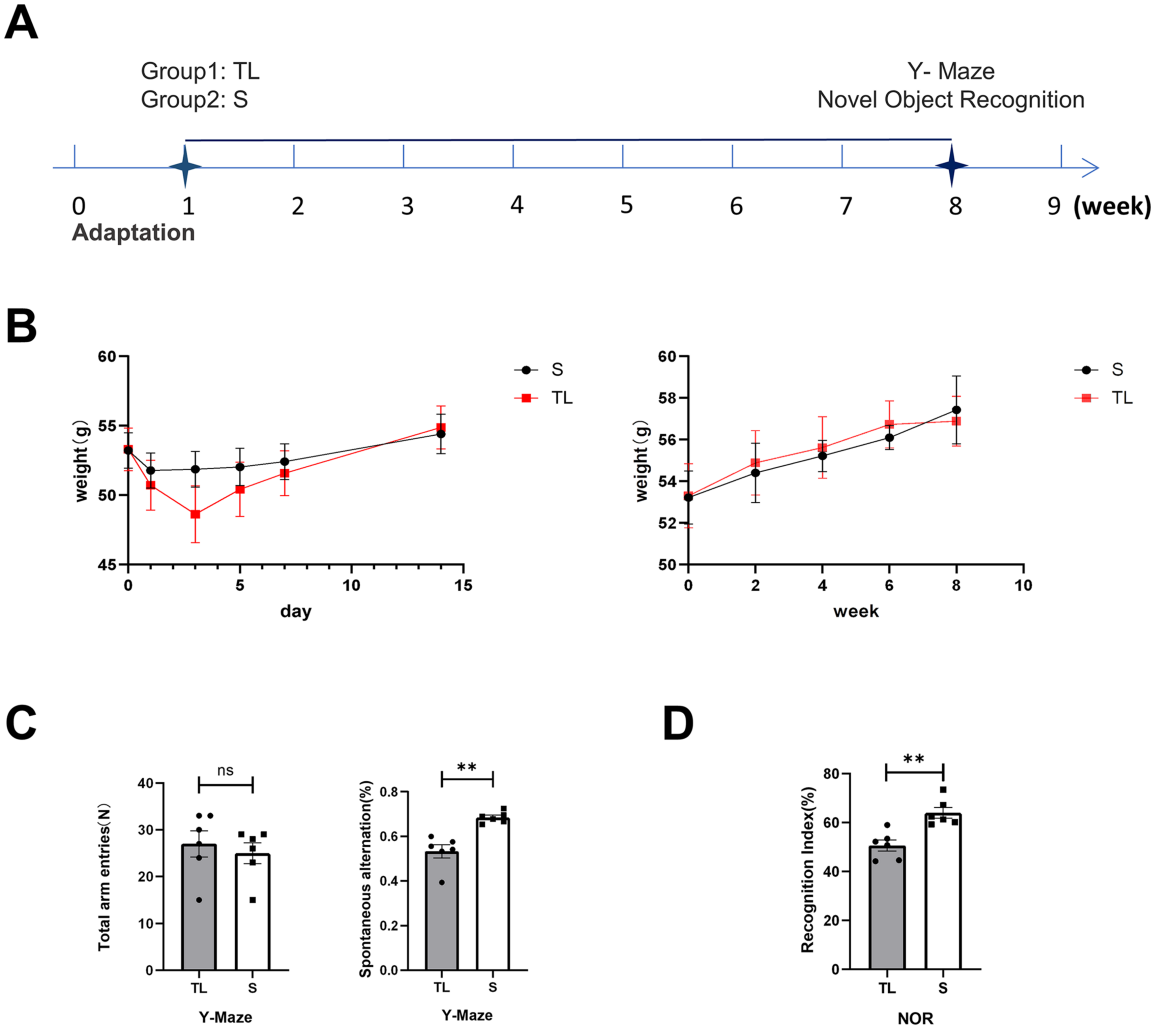
Effects of tooth loss on body weight and cognitive function in SAMP8 mice. **(A)** Experimental timelines, SAMP8 mice were tested for Y-maze and NOR 8 weeks after the animal model was established. **(B)** Changes in average body weight in each group during the initial day (day 0) and the 1st, 3rd, 5th, 7th, and 14th days and farther into the observation period of the experiment. The data are expressed as the mean ± SEM. **(C)** Performance of the Y-maze in SAMP8 mice, Mice were placed at the end of one of the three arms and the order in which they entered each arm within 5 min was recorded [S group (N = 6), TL group (N = 6), *p* = 0.0027; unpaired two-tailed t tests with Welch’s correction. For statistical details, see Supplementary Table 1]. **(D)** Performance of the NOR in SAMP8 mice, Mice were placed in the test box and removed after 5 min of free exploration. During the test phase, different objects were replaced to record the time the mice spent exploring the old and new objects [S group (N = 6), TL group (N = 6), *p* = 0.0019; unpaired two-tailed t tests. For statistical details, see Supplementary Table 2].

After bilateral upper molars extraction, the body weight of SAMP8 mice in the TL and S groups decreased after surgical trauma but returned to preoperative levels after 2 weeks (Figures 1A,B). This shows that the mice can recover from the trauma of the surgery and return to their preoperative body weight. During the 2 months following tooth extraction, SAMP8 mice showed a steady increase in body weight (Figure 1B). This shows that the mice can adapt to tooth loss and continue to gain weight, which suggests that the mice can maintain a healthy weight despite tooth loss.

### 3.2 Effects of tooth loss on brain and behavioral performance

**Figure 2:**
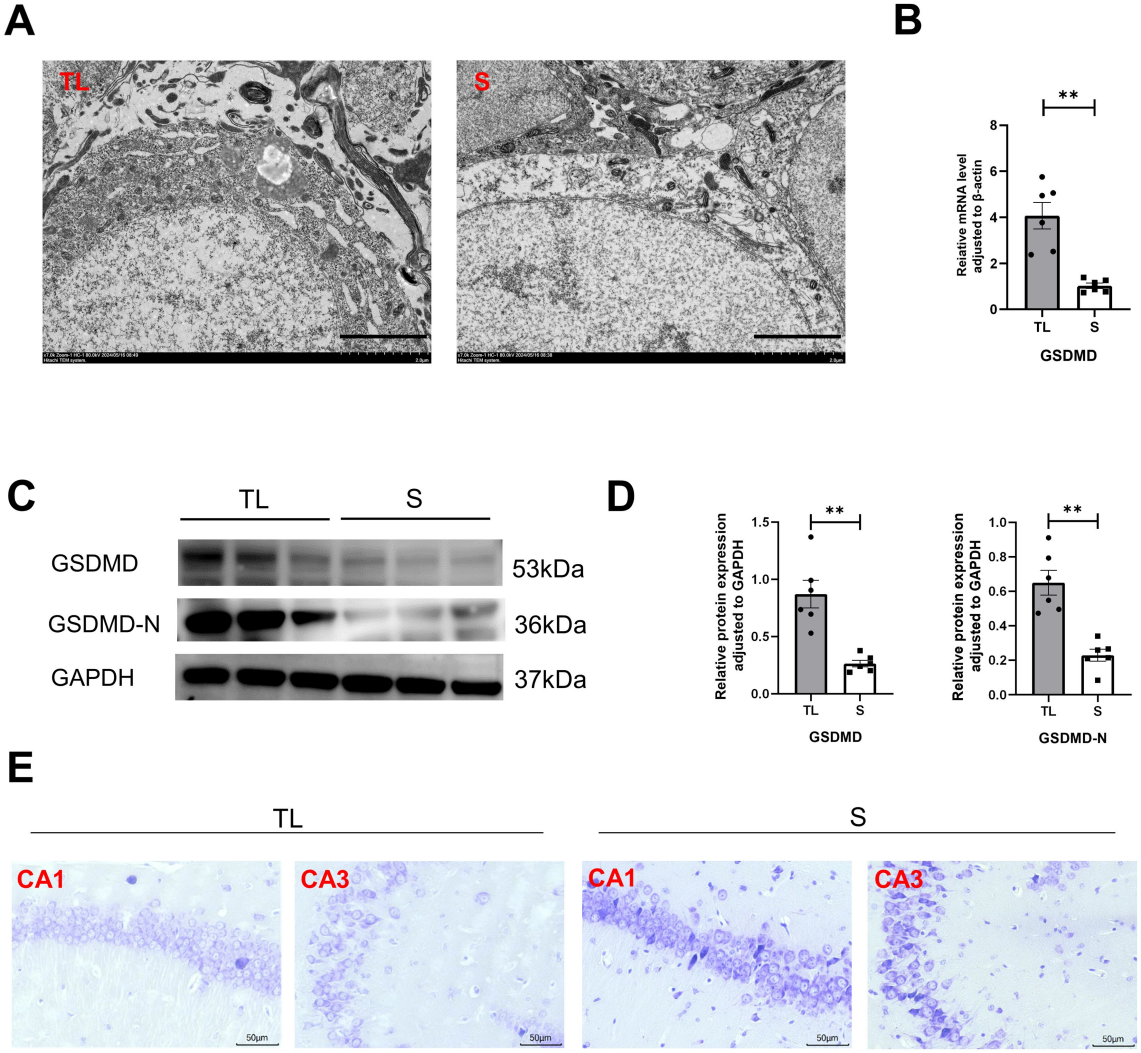
Tooth loss induces neuronal structural damage with upregulation of the pyroptosis target protein GSDMD in SAMP8 mice. **(A)** TEM images showing the state of hippocampal neurons in the TL and S groups (scale bar = 2.0 μm)**. (B)** Quantification of mRNA expression of GSDMD in S and TL normalized to *β*-actin [S group (N = 6), TL group (N = 6), *p* = 0.0029; unpaired two-tailed t tests with Welch’s correction. For statistical details, see Supplementary Table 3]. **(C)** Representative immunoblot images of GSDMD, GSDMD-N in protein extracts of hippocampal lysate samples from S and TL group SAMP8 mice. **(D)** Quantification of GSDMD, GSDMD-N in S and TL normalized to GAPDH (GSDMD, *p* = 0.0032, unpaired two-tailed t tests with Welch’s correction; GSDMD-N, *p* = 0.0011; unpaired two-tailed t tests with Welch’s correction). For statistical details, see Supplementary Tables 4.1, 4.2. **(E)** Representative pictographs showing Nissl staining (scale bar = 20 μm).

To explore the effects of loss of occlusal support on the behavioral performance of SAMP8 mice, we tested learning and memory abilities by Y-maze and NOR. The Y-maze was used to evaluate spatial working memory capacity in rodents, While NOR was used to assess their short-term memory capacity. The percentage of spontaneous alternation was significantly lower in the TL group of mice than in the S group (Figure 1C; *p* = 0.0022). At the same time, the recognition index of the TL group mice in the new object recognition experiment also significantly decreased (Figure 1D; *p* = 0.0019), suggesting cognitive dysfunction in SAMP8 mice after tooth extraction. In addition, neurons in the CA1 area of the hippocampus in the TL group were loosely arranged and there were fewer Nissl vesicles in the cytoplasm than in the control group (Figure 2E). Transmission electron microscopy results showed altered cell morphology after loss of occlusal support (Figure 2A).

### 3.3 Tooth loss exacerbates pyroptosis in SAMP8 mice

**Figure 3:**
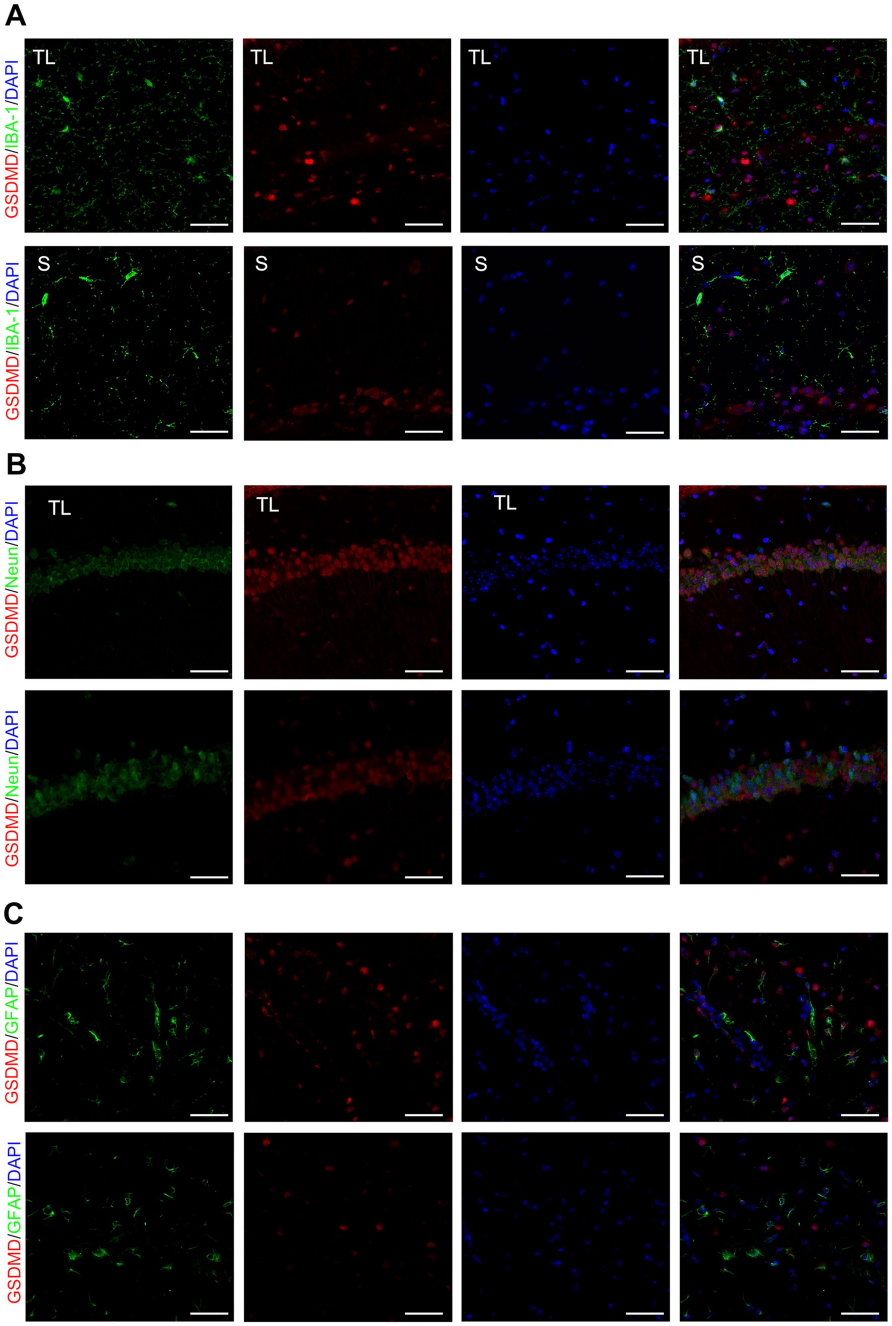
Co-localized expression of GSDMD in hippocampal microglia, neurons and astrocytes of SAMP8 mice. **(A–C)** Cell localization of GSDMD after tooth loss. Immunofluorescent double staining demonstrated the GSDMD mainly localized in the microglia [IBA-1, **(A)**], neurons [NeuN, **(B)**], and astrocytes [GFAP, **(C)**] after tooth loss (Scale bar = 50 um).

Tooth loss induced eminently neuronal damage, as evidenced by Nissl staining (Figure 2E), TEM (Figure 2A), and behavioral assays (Figures 1C,D), demonstrating simultaneous increases in the levels of GSDMD when compared with the S groups. We examined the cellular pyroptosis target protein GSDMD by immunofluorescence staining, qRT-PCR, and western blot. The results showed that the GSDMD mRNA expression was significantly up-regulated in the TL group compared with the S group, accompanied by changes in protein levels (Figures 2B–D). Next, we performed colocalization staining for GSDMD/microglial marker IBA-1 (Figure 3A), neuronal marker NeuN (Figure 3B), and astrocytic marker GFAP (Figure 3C) in the hippocampus tissues of the TL group and S group. The results showed that GSDMD expression was elevated and active in all cell types in the TL group. This suggests that tooth loss leads to increased expression of the pyroptosis markers, which further exacerbates the inflammatory response in the brain. In addition, excessive pyroptosis may lead to a further decline in cognitive performance.

### 3.4 Tooth loss aggravates cGAS-STING signaling in the brain of SAMP8 mice

**Figure 4:**
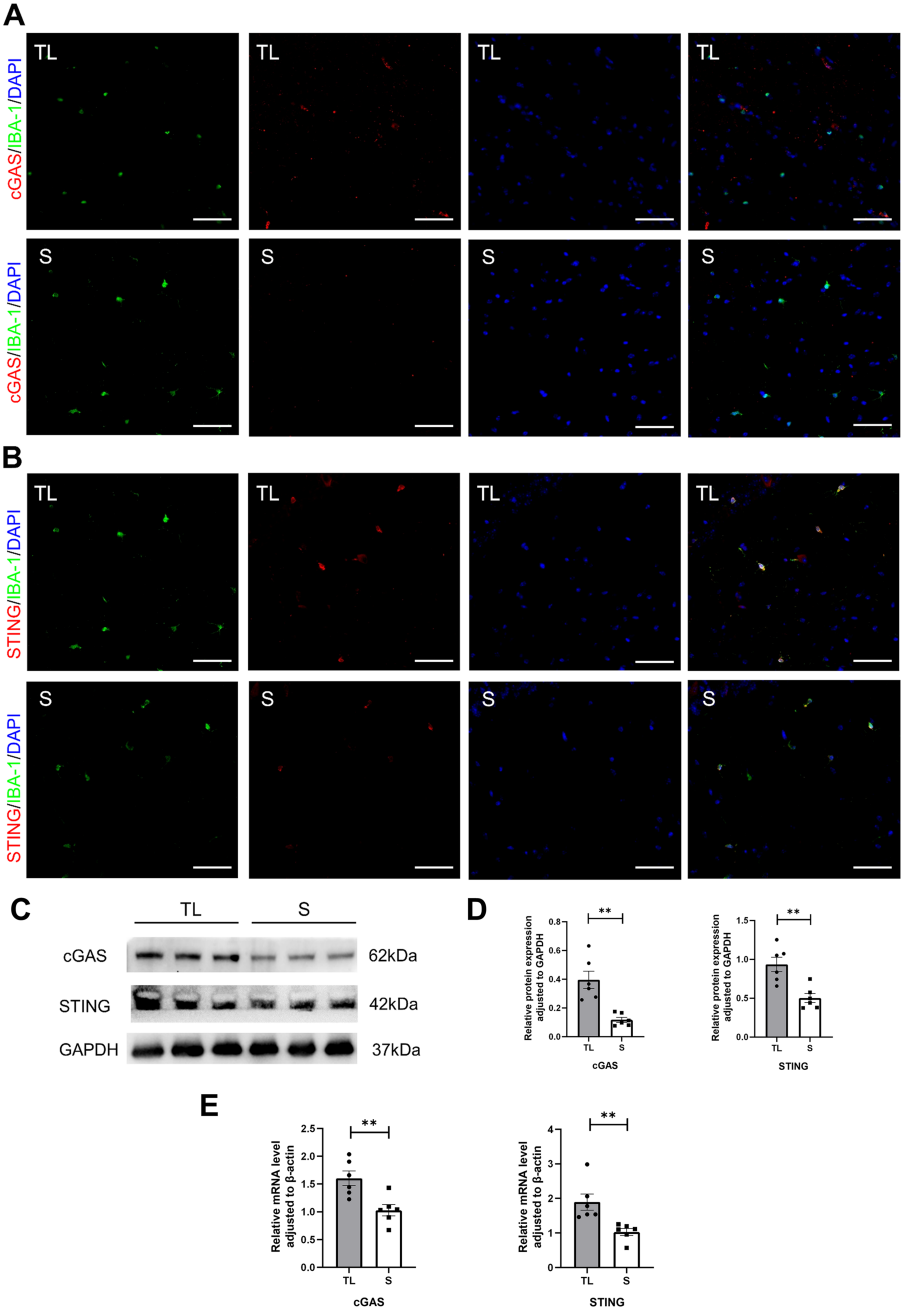
Tooth loss upregulates cGAS-STING expression in SAMP8 mice. **(A,B)** Cell localization of cGAS and STING after tooth loss. Immunofluorescent double staining demonstrated the cGAS and STING mainly localized in the microglia after tooth loss (scale bar = 50 um). **(C)** Representative immunoblot images of cGAS, STING in protein extracts of hippocampal lysate samples from S and TL group SAMP8 mice. **(D)** Quantification of cGAS, STING in S and TL normalized to GAPDH (cGAS, *p* = 0.0045, unpaired two-tailed t tests with Welch’s correction; STING, *p* = 0.0027, unpaired two-tailed t tests.) For statistical details, see Supplementary Tables 5.1, 5.2. **(E)** Quantification of mRNA expression of cGAS, STING in S and TL normalized to β-actin (cGAS, *p* = 0.0061, unpaired two-tailed t tests; STING, *p* = 0.0022; Mann–Whitney U test). For statistical details, see Supplementary Tables 6.1, 6.2.

**Figure 5:**
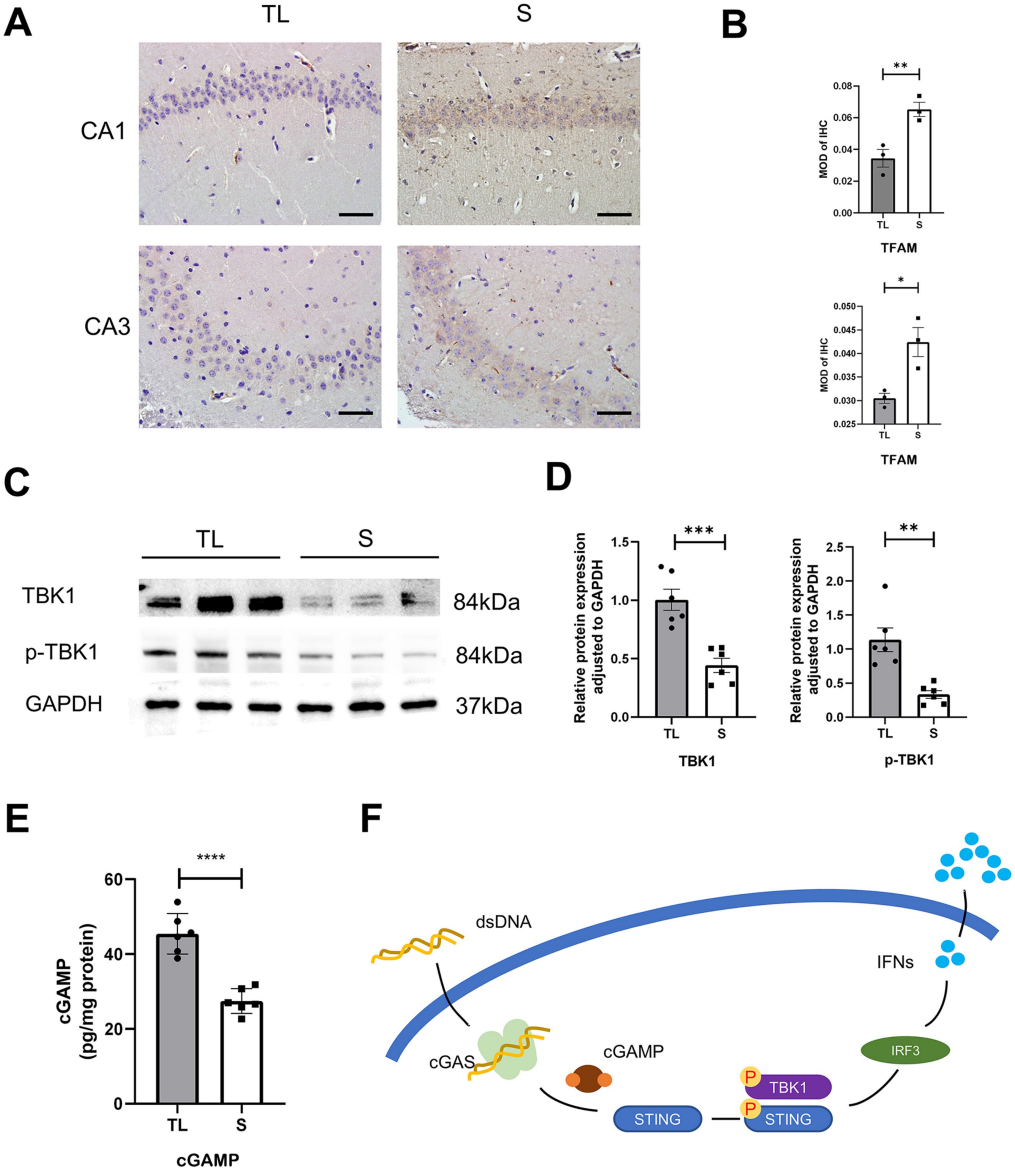
Tooth loss activates the cGAS-STING upstream and downstream pathways in the hippocampus of SAMP8 mice. **(A)** Typical immunohistochemical staining for TFAM in the CA1 and CA3 regions of the hippocampus at diverse groups after tooth loss. (Scale bar = 50 um) **(B)** Quantification of TFAM MOD in the S and TL groups. (CA1, *p* = 0.0016, unpaired two-tailed t tests; CA3, *p* = 0.0219; unpaired two-tailed t tests.) For statistical details, see Supplementary Tables 7.1, 7.2. **(C)** Representative immunoblot images of TBK1, p-TBK1 in protein extracts of hippocampal lysate samples from S and TL group SAMP8 mice. **(D)** Quantification of TBK1, p-TBK1 in S and TL normalized to GAPDH (TBK1, *p* = 0.0004, unpaired two-tailed t tests; p-TBK1, *p* = 0.0045; unpaired two-tailed t tests with Welch’s correction). For statistical details, see Supplementary Tables 8.1, 8.2. **(E)** Quantitative analysis of ELISA expression of cGAMP in the S, Tlgroup (*p* = 0.0001; unpaired two-tailed t tests). For statistical details, see Supplementary Table 9. **(F)** A schematic showing the downstream initiation of the microglia cGAS-STING axis upon recognition of dsDNA.

cGAS and STING expression in hippocampal microglia was assessed by immunofluorescence staining. As shown in Figures 4A,B. Immunofluorescent double staining showed that the cGAS and STING are more frequently expressed in the activated microglia/macrophages (IBA-1+) after loss of occlusal support. This also indicates that the number of cGAS- or STING-positive cells in hippocampal microglia was significantly higher in the TL group than in the S group. Then, the qRT-PCR and western blot were used to detect the mRNA and protein expressions, and the results were consistent with those of immunofluorescence staining (Figures 4C–E). As shown in Figure 5F, this figure illustrates the classic activation process of the cGAS-STING signaling pathway. cGAS, activated by dsDNA, catalyzes the production of cyclic GMP-AMP (cGAMP). As a second messenger, cGAMP binds to the stimulator of interferon genes (STING), thereby activating STING. Activated STING recruits and activates TANK-binding kinase 1 (TBK1), which plays a key role in immune responses. In this study, the decrease of TFAM expression in the hippocampus following tooth loss indicates the potential presence of mitochondrial impairment and excessive accumulation of mtDNA (Figures 5A,B). Additionally, studies have found that, compared to the S group, the levels of TBK1, p-TBK1, and cGAMP in the hippocampus were significantly higher in the TL group. This suggests that the cGAS-STING pathway is more active after tooth loss (Figures 5C–E). Excessive activation of the cGAS-STING axis is highly likely to originate from the abnormal accumulation of mtDNA following loss of occlusal support.

### 3.5 Microglia pyroptosis was aggravated by upregulation of the cGAS-STING pathway in BV2 cells

**Figure 6:**
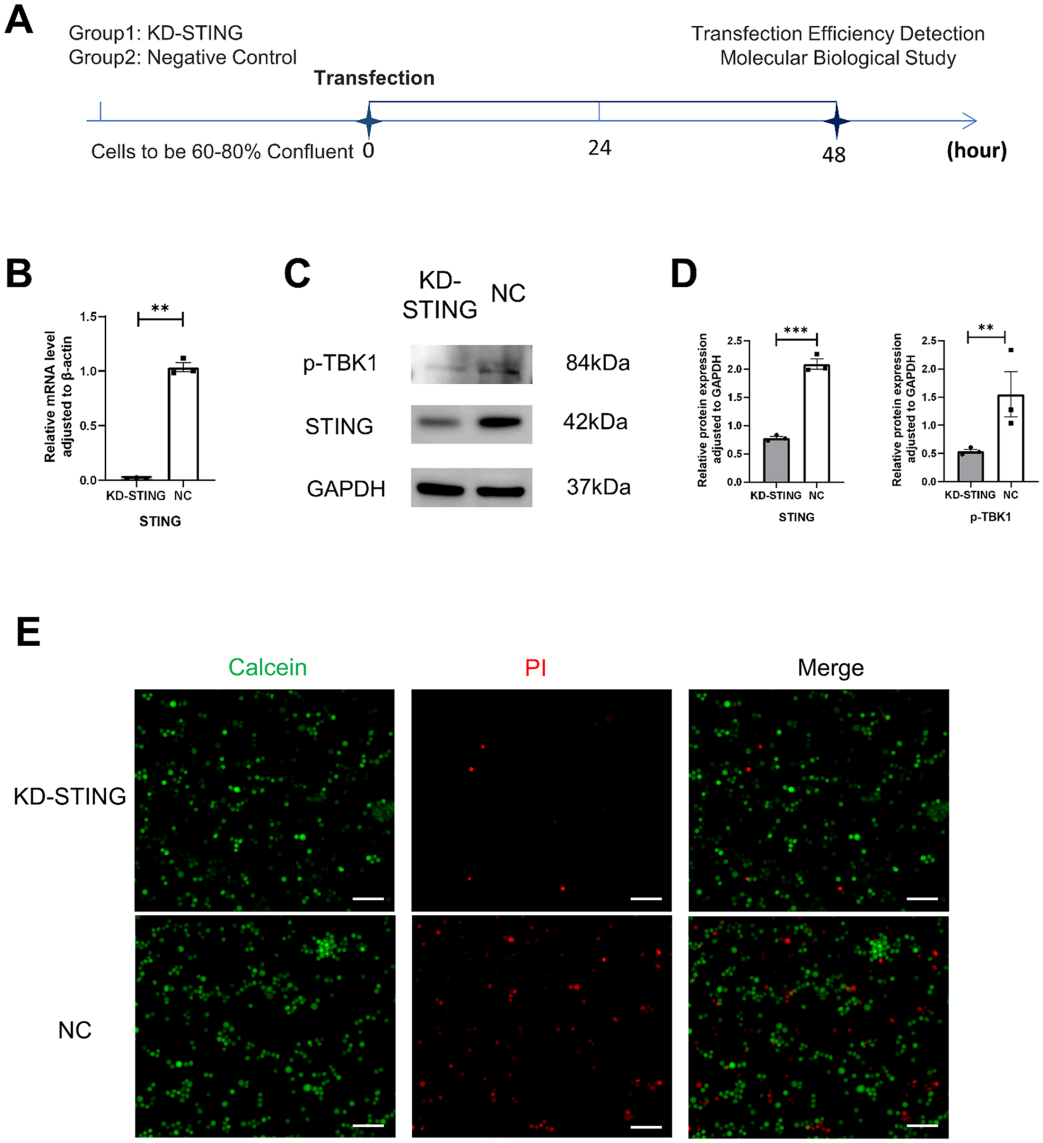
Knockdown of STING in BV2 cells rescued its death. **(A)** Experimental timelines, BV2 Cells were 60–80% confluent to start transfection, and subsequent assays were performed after 48 h. **(B)** Quantification of mRNA expression after STING knockdown normalized to β-actin (*p* = 0.0017, unpaired two-tailed t tests with Welch’s correction) For statistical details, see Supplementary Table 10. **(C)** Representative immunoblot images of p-TBK1, STING in a diverse group of BV2 cell lysate protein extracts. **(D)** Quantification of p-TBK1, STING in NC (N = 3)and KD-STING (N = 3) normalized to GAPDH (p-TBK1, *p* = 0.0011, unpaired two-tailed t tests with Welch’s correction; STING, *p* = 0.0001, unpaired two-tailed t tests). For statistical details, see Supplementary Tables 11.1, 11.2. **(E)** Fluorescence staining map of BV2 cells alive and dead after STING knockdown (Scale bar = 200 um).

**Figure 7:**
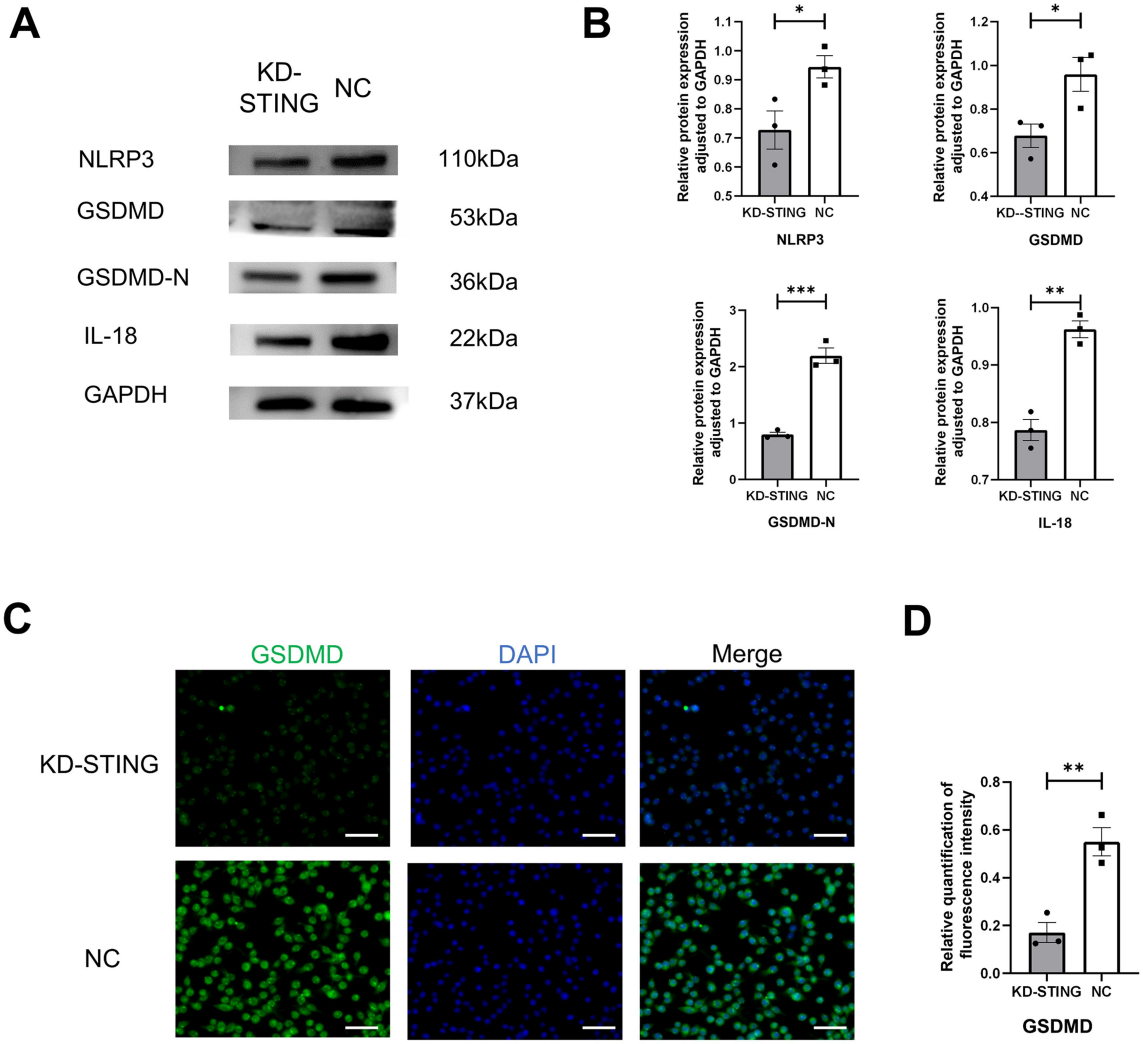
Knockdown of STING in BV2 cells inhibits GSDMD-induced cellular pyroptosis. **(A)** Representative immunoblot images of NLRP3, GSDMD, GSDMD-N, IL-18 in a diverse group of BV2 cell lysate protein extracts. **(B)** Quantification of NLRP3, GSDMD, GSDMD-N, IL-18 in NC (N = 3) and KD-STING (N = 3) normalized to GAPDH (NLRP3, *p* = 0.0465, unpaired two-tailed t tests, GSDMD *p* = 0 0.0407, unpaired two-tailed t tests, GSDMD-N *p* = 0.0006, unpaired two-tailed t tests, IL-18 *p* = 0.0017, unpaired two-tailed t tests). For statistical details, see Supplementary Tables 12.1–12.4. **(C)** Typical immunohistochemical staining for GSDMD at diverse groups after STING knockdown. Scale bar = 50 μm. **(D)** Quantification of GSDMD fluorescence intensity in the NC and KD-STING groups. *p* = 0.0063, unpaired two-tailed t tests. For statistical details, see Supplementary Table 13.

To further observe the cGAS-STING signaling pathway and pyroptosis, the BV2 cells model was used *in vitro* (Figure 6A). As shown in Figure 6B, the qRT-PCR results showed that the use of STING siRNA efficiently reduced the expression of STING. Additionally, we also demonstrated that the protein levels of STING, p-TBK1, NLRP3, GSDMD, GSDMD-N, and IL-18 were markedly reduced in comparison with the si-NC groups (Figures 6C,D, 7A,B). Concurrently, GSDMD fluorescence staining results in BV2 cells showed the same trend (Figures 7C,D). What’s more, the delivery of STING siRNA also remarkably restrained cell apoptosis to some extent and decreased the counts of dying BV2 cells in the si-STING groups, as demonstrated by Calcein/PI and immunofluorescence staining (Figure 6E). The above results suggest that tooth loss can induce hippocampal microglia to exhibit pyroptotic features, and this process may be involved in pathological damage, which is possibly mediated by the increased expression of GSDMD-N and the increased number of inflammatory factor-positive cells. In addition, the cGAS-STING signaling pathway plays a crucial role in hippocampal microglia pyroptosis, which may contribute to the cognitive deficits observed in the TL group.

## 4 Discussion

AD is a severe neurodegenerative disease, which is currently the most predominant type of dementia-related disease, first proposed by the German physician Alois Alzheimer in 1906, and later formally named by Kraeplin (Hippius and Neundörfer, 2003; Gottfries, 1988). The pathology of AD is complex and varied, with neuronal loss, synaptic deficits (e.g., synaptic loss and prominent plasticity defects), extracellular amyloid beta (Aβ) deposits forming amyloid plaques, and abnormally phosphorylated Tau proteins forming intracellular neurofibrillary tangles, all of which are prevalent in patients with AD (Scheltens et al., 2016; Bondi et al., 2017). SAMP8 mice have been utilised as a prominent animal model of ageing and dementia due to their manifestation of hallmark biological characteristics associated with ageing, including cognitive decline and Aβ aggregation in the brain. These characteristics are analogous to those observed in human AD (Liu et al., 2020; Griñán-Ferré et al., 2018; Cheng et al., 2014). This study aimed to observe the histopathological changes in the hippocampal region induced by tooth loss in SAMP8 mice and their effect on cognitive dysfunction and subsequent *in vitro* validation of the molecular mechanisms.

In the past few decades, despite the emergence of several theories on the development of AD, AD certainly arises and develops as a result of a combination of factors. Recent studies have identified loss of occlusal support due to tooth loss as a risk factor for AD (Nakamura et al., 2021; Singhrao et al., 2014). In this experiment, SAMP8 mice in the TL group performed worse than those in the S group in both the Y-maze spontaneous alternation experiment and the new object recognition experiment, showing cognitive decline. Moreover, the aforementioned results are consistent with the findings of previous studies.

Extensive research on regulatory cell death in AD has revealed mounting evidence for a close relationship between pyroptosis and the occurrence, development, and prognosis of AD (Wang et al., 2023; Han et al., 2020). Sebastiaan Moonen et al. investigated multiple cases of symptomatic AD and pathologically defined preclinical AD and found that expression of cleaved GSDMD, the pyroptosis effector protein, was increased in the brains of AD patients compared to controls, particularly in microglia (Moonen et al., 2023). In this study, a significant increase in the number of GSDMD-positive cells in the hippocampus of SAMP8 mice was observed in NeuN+ cells, GFAP+ cells, and especially in IBA-1 + cells after tooth loss. TEM results showed that abnormalities of altered cellular morphology were also observed in the TL group. Meanwhile, our findings demonstrated that the mRNA and protein expression levels of GSDMD and GSDMD-N were increased in the hippocampus of the TL group SAMP8 mice, suggesting that the pyroptosis pathway may be activated after tooth loss.

Microglia are the resident immune cells in charge of immune defense and inflammation modulation in the central nervous system (CNS) (Ding et al., 2022). Their excessive activation leads to imbalances in the homeostasis of the brain and is thought to be closely associated with neurodegenerative diseases (Qiu et al., 2023). The cGAS-STING signaling pathway is one of the fundamental mechanisms of host defense in organisms by establishing an effective immune response and inducing a series of genes related to inflammation to be expressed (Decout et al., 2021). In recent years, researchers at home and abroad have shown that activation of the microglia cGAS-STING pathway plays a wide range of roles in neurodegenerative diseases (Decout et al., 2021; Gulen et al., 2023). In the present study, immunofluorescence staining showed a significant increase in the number of cGAS- and STING-positive microglia in the TL group. In addition, the western blots results showed that the downstream targeting factors of STING, TBK1, and *p*-TBK1 were upregulated in the TL group compared with the S group, while the ELISA results also illustrated the upregulation of cGAMP expression in the TL group. The above results suggest active expression of the cGAS-STING pathway after loss of occlusal support.

Available evidence confirms that cGAS or STING inhibition significantly attenuates neuroinflammation and brain damage (Carling et al., 2024). STING knockdown in microglia inhibited TNF-*α* release and significantly reduced the expression of factors such as iNO (Zhang et al., 2024). The antagonist of cGAS, A151, effectively reduced the expression of pyroptosis-associated molecules and diminished cell death (Li et al., 2020). In this study, siRNA was used to knock down the expression of STING in BV2 cells. We found that the knockdown of STING effectively rescued BV2 cells from exhibiting pyroptotic features, suggesting that the expression of STING-related genes plays an important role in the pyroptosis of BV2 cells. However, the mechanism underpinning STING-modulated microglial pyroptosis is still not fully elucidated and requires further investigation.

Mitochondria, a two-membrane organelle found in most eukaryotic cells, are the energy-producing structure of the cell and the main site of aerobic respiration, known as the “powerhouse” (Monzel et al., 2023). Abnormal mitochondrial function, mitochondrial damage, and autophagy have been reported to occur after tooth loss, as well as the accompanying gradual accumulation of mitochondrial DNA (mtDNA) mutations (Wang et al., 2022; Katano et al., 2020). Mitochondrial DNA is a circular dsDNA molecule in the cytoplasm of the cell, located in the mitochondria outside the nucleus. When mtDNA is abnormally over-accumulated in the cell, it can bind to relevant recognition receptors. Mitochondrial transcription factor A (TFAM) is a protein encoded by nuclear genes that are synthesized in the cytoplasm and then translocated to mitochondria to perform its function. TFAM is reported to be a key protein in maintaining the functional stability of mtDNA (Song et al., 2024). When TFAM is absent, the mitochondrial structure is impaired, resulting in the abnormal accumulation of mtDNA in the cytoplasm, which is recognized by cGAS and activates the cGAS-STING signaling pathway (Liu et al., 2024). In the study, immunohistochemical staining showed decreased expression of TFAM in the hippocampus of SAMP8 mice in the TL group, suggesting increased levels of mitochondrial stress and abnormal mitochondrial homeostasis. The results of the present study suggest that SAMP8 mice may activate the cGAS-STING pathway through an aberrant accumulation of mtDNA after the loss of occlusal support. This, in turn, triggers a neuroinflammatory state and thus exacerbates cognitive behavioral deficits. However, further investigation is required into the specific mechanisms of STING activation following tooth loss.

Based on this experiment, we hypothesized that occlusal support loss due to tooth loss may cause cognitive dysfunction by promoting mtDNA overaccumulation in the hippocampal region of SAMP8 mice in the Alzheimer’s disease model, up-regulating the cGAS/STING pathway, stimulating downstream immune responses, and triggering microglial cell pyroptosis.

This study has the limitation that the upstream targeted molecules of the mtDNA-cGAS-STING pathway and their activation mechanisms were not explored in depth. Therefore, future studies are needed to investigate the mechanisms of changes to dsDNA and their subsequent influence on cognitive function after tooth loss.

## Data Availability

The raw data supporting the conclusions of this article will be made available by the authors, without undue reservation.
