# Supplementary material for: Tooth loss impairs cognitive function in SAMP8 mice by aggravating pyroptosis of microglia via the cGAS/STING pathway
Source: Front Aging Neurosci. 2025 Aug 22;17:1628520. doi: 10.3389/fnagi.2025.1628520 (PMC12411515; doi:10.3389/fnagi.2025.1628520)
Supplement: Supplementary file 1 [file Data_Sheet_1.docx]

Supplementary Material

# Supplementary Tables

**TABLE 1. 1** Total arm entries in TL and S group (n=6)

|  | **TL** | **S** |
| --- | --- | --- |
| 1 | 33 | 23 |
| 2 | 15 | 29 |
| 3 | 33 | 15 |
| 4 | 30 | 29 |
| 5 | 24 | 26 |
| 6 | 27 | 28 |
| Mean±SEM | 27±2.793 | 25±2.206 |

**TABLE 1. 2** Spontaneous Alternation in TL and S group (n=6)

|  | **TL** | **S** |
| --- | --- | --- |
| 1 | 0.576 | 0.696 |
| 2 | 0.600 | 0.724 |
| 3 | 0.394 | 0.667 |
| 4 | 0.533 | 0.690 |
| 5 | 0.542 | 0.654 |
| 6 | 0.556 | 0.679 |
| Mean±SEM | 0.533±0.030 | 0.685±0.010 |

**TABLE 2.** Recognition Index in TL and S group (n=6)

|  | **TL** | **S** |
| --- | --- | --- |
| 1 | 59.0 | 60.2 |
| 2 | 50.8 | 61.3 |
| 3 | 53.4 | 59.3 |
| 4 | 52.2 | 73.5 |
| 5 | 44.2 | 62.5 |
| 6 | 44.6 | 67.2 |
| Mean±SEM | 50.7±2.294 | 64.00±2.209 |

**TABLE 3.** Relative mRNA level adjusted to β-actin of GSDMD for RT-PCR (n=6)

|  | **TL** | **S** |
| --- | --- | --- |
| 1 | 5.054 | 0.7324 |
| 2 | 5.758 | 1.263 |
| 3 | 4.944 | 0.773 |
| 4 | 2.518 | 1.157 |
| 5 | 2.374 | 1.392 |
| 6 | 3.769 | 0.867 |
| Mean±SEM | 4.069±0.5761 | 1.031±0.1130 |

**TABLE 4.1** Relative protein expression adjusted to GAPDH of GSDMD for WB (n=6)

|  | **TL** | **S** |
| --- | --- | --- |
| 1 | 0.906 | 0.378 |
| 2 | 0.736 | 0.242 |
| 3 | 0.530 | 0.315 |
| 4 | 1.371 | 0.264 |
| 5 | 0.991 | 0.190 |
| 6 | 0.697 | 0.205 |
| Mean±SEM | 0.872±0.120 | 0.266±0.029 |

**TABLE 4.2** Relative protein expression adjusted to GAPDH of GSDMD-N for WB (n=6)

|  | **TL** | **S** |
| --- | --- | --- |
| 1 | 0.911 | 0.084 |
| 2 | 0.793 | 0.259 |
| 3 | 0.473 | 0.341 |
| 4 | 0.678 | 0.266 |
| 5 | 0.548 | 0.210 |
| 6 | 0.495 | 0.215 |
| Mean±SEM | 0.650±0.072 | 0.229±0.035 |

**TABLE 5.1** Relative protein expression adjusted to GAPDH of cGAS for WB (n=6)

|  | **TL** | **S** |
| --- | --- | --- |
| 1 | 0.335 | 0.080 |
| 2 | 0.256 | 0.097 |
| 3 | 0.276 | 0.076 |
| 4 | 0.632 | 0.166 |
| 5 | 0.511 | 0.175 |
| 6 | 0.367 | 0.112 |
| Mean±SEM | 0.396±0.060 | 0.118±0.018 |

**TABLE 5.2** Relative protein expression adjusted to GAPDH of STING for WB (n=6)

|  | **TL** | **S** |
| --- | --- | --- |
| 1 | 0.835 | 0.380 |
| 2 | 0.656 | 0.497 |
| 3 | 0.743 | 0.576 |
| 4 | 1.253 | 0.749 |
| 5 | 1.073 | 0.439 |
| 6 | 1.052 | 0.376 |
| Mean±SEM | 0.936±0.093 | 0.503±0.058 |

**TABLE 6.1** Relative mRNA level adjusted to β-actin of cGAS for RT-PCR (n=6)

|  | **TL** | **S** |
| --- | --- | --- |
| 1 | 1.227 | 1.044 |
| 2 | 1.659 | 1.029 |
| 3 | 2.037 | 1.432 |
| 4 | 1.347 | 0.899 |
| 5 | 1.444 | 0.672 |
| 6 | 1.903 | 1.096 |
| Mean±SEM | 1.603±0.131 | 1.029±0.102 |

**TABLE 6.2** Relative mRNA level adjusted to β-actin of STING for RT-PCR (n=6)

|  | **TL** | **S** |
| --- | --- | --- |
| 1 | 1.462 | 0.954 |
| 2 | 1.778 | 1.253 |
| 3 | 2.061 | 1.191 |
| 4 | 1.537 | 1.099 |
| 5 | 1.537 | 0.569 |
| 6 | 2.986 | 1.113 |
| Mean±SEM | 1.894±0.2363 | 1.030±0.1009 |

**TABLE 7.1** MOD values for immunohistochemistry of TFAM CA1 (n=3)

|  | **TL** | **S** |
| --- | --- | --- |
| 1 | 0.037 | 0.058 |
| 2 | 0.043 | 0.064 |
| 3 | 0.024 | 0.074 |
| Mean±SEM | 0.035±0.004 | 0.065±0.003 |

**TABLE 7.2** MOD values for immunohistochemistry of TFAM CA3 (n=3)

|  | **TL** | **S** |
| --- | --- | --- |
| 1 | 0.031 | 0.048 |
| 2 | 0.029 | 0.037 |
| 3 | 0.032 | 0.043 |
| Mean±SEM | 0.030±0.001 | 0.042±0.003 |

**TABLE 8.1** Relative protein expression adjusted to GAPDH of TBK1 for WB (n=6)

|  | **TL** | **S** |
| --- | --- | --- |
| 1 | 0.763 | 0.272 |
| 2 | 1.252 | 0.283 |
| 3 | 1.288 | 0.589 |
| 4 | 1.019 | 0.595 |
| 5 | 0.839 | 0.538 |
| 6 | 0.866 | 0.383 |
| Mean±SEM | 1.004±0.091 | 0.4433±0.061 |

**TABLE 8.2** Relative protein expression adjusted to GAPDH of p-TBK1 for WB (n=6)

|  | **TL** | **S** |
| --- | --- | --- |
| 1 | 0.822 | 0.420 |
| 2 | 1.023 | 0.191 |
| 3 | 0.771 | 0.175 |
| 4 | 1.923 | 0.536 |
| 5 | 1.274 | 0.387 |
| 6 | 1.004 | 0.298 |
| Mean±SEM | 1.136±0.173 | 0.334±0.057 |

**TABLE 9.** Relative expression of cGAMP for ELISA (n=6)

|  | **TL** | **S** |
| --- | --- | --- |
| 1 | 44.6 | 30.7 |
| 2 | 45.7 | 26.6 |
| 3 | 53.9 | 25.9 |
| 4 | 38.9 | 22.7 |
| 5 | 48.8 | 27.0 |
| 6 | 40.8 | 31.8 |
| Mean±SEM | 45.45±2.221 | 27.45±1.359 |

**TABLE 10.** Relative mRNA level adjusted to β-actin of STING for RT-PCR (n=3)

|  | **KD-STING** | **NC** |
| --- | --- | --- |
| 1 | 0.017 | 0.986 |
| 2 | 0.016 | 1.015 |
| 3 | 0.022 | 1.119 |
| Mean±SEM | 0.018±0.002 | 1.040±0.042 |

**TABLE 11.1** Relative protein expression adjusted to GAPDH of STING for WB (n=3)

|  | **KD-STING** | **NC** |
| --- | --- | --- |
| 1 | 0.772 | 1.987 |
| 2 | 0.732 | 2.265 |
| 3 | 0.831 | 2.015 |
| Mean±SEM | 0.778±0.029 | 2.089±0.088 |

**TABLE 11.2** Relative protein expression adjusted to GAPDH of p-TBK1 for WB (n=3)

|  | **KD-STING** | **NC** |
| --- | --- | --- |
| 1 | 0.608 | 1.037 |
| 2 | 0.484 | 1.274 |
| 3 | 0.526 | 2.336 |
| Mean±SEM | 0.5392±0.037 | 1.549±0,400 |

**TABLE 12.1** Relative protein expression adjusted to GAPDH of NLRP3 for WB (n=3)

|  | **KD-STING** | **NC** |
| --- | --- | --- |
| 1 | 0.833 | 0.936 |
| 2 | 0.607 | 1.015 |
| 3 | 0.742 | 0.882 |
| Mean±SEM | 0.727±0.066 | 0.945±0.038 |

**TABLE 12.2** Relative protein expression adjusted to GAPDH of GSDMD for WB (n=3)

|  | **KD-STING** | **NC** |
| --- | --- | --- |
| 1 | 0.572 | 0.803 |
| 2 | 0.738 | 1.046 |
| 3 | 0.723 | 1.027 |
| Mean±SEM | 0.678±0.053 | 0.959±0.078 |

**TABLE 12.3** Relative protein expression adjusted to GAPDH of GSDMD-N for WB (n=3)

|  | **KD-STING** | **NC** |
| --- | --- | --- |
| 1 | 0.765 | 2.031 |
| 2 | 0.749 | 2.462 |
| 3 | 0.884 | 2.095 |
| Mean±SEM | 0.799±0.043 | 2.196±0.134 |

**TABLE 12.4** Relative protein expression adjusted to GAPDH of IL-18 for WB (n=3)

|  | **KD-STING** | **NC** |
| --- | --- | --- |
| 1 | 0.819 | 0.963 |
| 2 | 0.786 | 0.987 |
| 3 | 0.755 | 0.937 |
| Mean±SEM | 0.787±0.018 | 0.963±0.015 |

**TABLE 13.** MOD values for immunofluorescence of GSDMD (n=3)

|  | **KD-STING** | **NC** |
| --- | --- | --- |
| 1 | 0.125 | 0.462 |
| 2 | 0.135 | 0.526 |
| 3 | 0.254 | 0.663 |
| Mean±SEM | 0.171±0.041 | 0.550±0.059 |
