# Supplementary material for: Tooth loss impairs cognitive function in SAMP8 mice by aggravating pyroptosis of microglia via the cGAS/STING pathway
Source: Front Aging Neurosci. 2025 Aug 22;17:1628520. doi: 10.3389/fnagi.2025.1628520 (PMC12411515; doi:10.3389/fnagi.2025.1628520)

Tooth Loss Impairs Cognitive Function by aggravating pyroptosis of microglia via the cGAS/STING Pathway

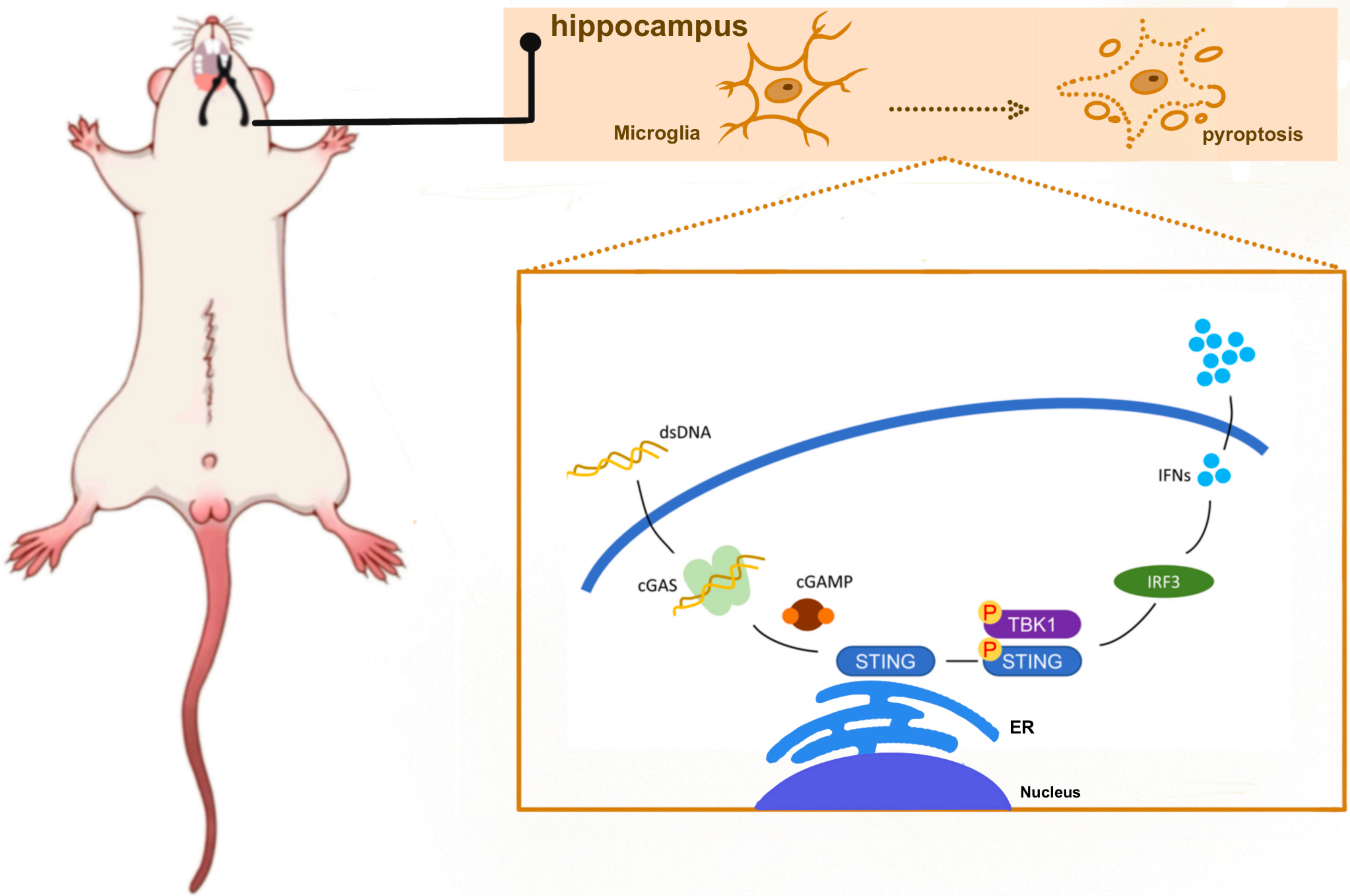

Supplement: Supplementary file 2 [file Image_1.pdf]
